# Towards a national policy on nursing education and training: an imperative framework for integrating nursing education within South Africa’s post-school education system

**DOI:** 10.1186/s12912-024-01880-6

**Published:** 2024-04-28

**Authors:** Vhothusa Edward Matahela, Nonhlanhla Jabulile Makhanya

**Affiliations:** 1 National Department of Health, Pretoria, South Africa; 2 Department of Health Studies, University of South Africa, Pretoria, South Africa

**Keywords:** Nursing, Nursing education, Public policy, Policy development, Education, South Africa

## Abstract

The aim of this study article is to present an analysis of the first national policy framework, which provides a coherent approach to integrating nursing education into a newly defined band for higher education programmes in South Africa. The significance of this policy framework is ensuring the seamless transition from legacy nursing programmes to NQF-registered nursing programmes. It explores the agenda-setting process, analyses the prevailing context and outlines the rationale for the policy. Walt and Gilson’s policy triage analysis process outlines the key elements of the policy development process. Drawing upon Tarlov’s two-phased public policy development process, the article outlines the steps completed in the policy development process. Recommendations are proposed to expand access, improve quality and diversify the provisioning of nursing education and training in South Africa.

## Introduction

Nurses and midwives in South Africa comprise more than 56% of the total health workforce [1]. They are central in addressing the quadruple burden of diseases: the coinciding epidemics of HIV, AIDS and tuberculosis; high maternal and child mortality; noncommunicable diseases; and violence and injuries [2]. South Africa’s healthcare system recognizes nurses and midwives as the cornerstone of health service delivery. They are often the first and sometimes the sole interface with healthcare users, particularly at the primary healthcare level. For example, the country has the highest number of people living with HIV [3], thus an increase in the demand for an adequate nursing and midwifery workforce to strengthen the public health sector’s response to HIV and AIDS, including initiation of antiretroviral therapy and its management at primary health care level. In addition, the Covid-19 epidemic has had an unprecedent reversal of gains in the reduction of country’s child and maternal mortality rates, which are now well behind the Sustainable Developmental Goals. Currently, the country’s institutional maternal mortality (iMMR) stands at 120 per 100,000 live births, the infant mortality rate at 25 deaths per 1,000 live births, and the institutional neonatal death rate is 12 per 1,000 live births. There is a need for massification of nurses and midwives with required skills mix for the country to combat high maternal and neonatal mortality rates [4]. The dynamic nature of health system demands a high level of competence to meet current and future population health needs, which underscores the importance of well-prepared clinical nurse practioners. The government holds the directive to harmonise nursing programmes with service delivery and clinical competencies to conform with national practice standards [5]. Nurse training has historically been offered under interim arrangement and not fully integrated into the post-school education system. The advent of the new National Qualifications Framework (NQF) provided an opportunity for nursing education to be integrated into the post-school education system. Hence, a policy was needed to align nursing education with post-school education legislative prescripts to foster transformative, high-quality education for nurses to effectively produce safe and competent practitioners, thus addressing population health needs [6]. This is referred to as the National Policy on Nursing Education and Training. This policy should become the overarching framework that ensures coherent implementation of NQF-aligned nursing programmes by all providers of nursing education programmes [7, 8]. The policy needed to undergo a robust policy development process, extensive stakeholder engagement and validation, political engagement, along with an integrated socio-economic assessment process for successful implementation. This article presents a rigorous analysis of this first national policy framework. The framework aims to ensure an inclusive and coherent approach to nursing education and training with recommendations to facilitate seamless adoption thereof.

## Background

Section 52(a-d) of the National Health Act (Act 61 of 2003) mandate that the Minister of Health provide a consistent supply of healthcare professionals with the requisite skill mix to meet current and future health demands [9]. Cognizant of the centrality of nursing and midwifery services in ensuring a responsive health system, Minister of Health Dr A. Motsoaledi, in 2011, convened a National Nursing Summit that aimed to address the challenges facing the nursing profession [10]. Through deliberate engagement modalities, including explicit political support, presentations, dialogues and sharing experiences from community of practices, the Summit allowed nurses to reflect on issues affecting their profession and the health system and anticipated reforms. This approach culminated in a Nursing Compact, thus providing a summary of resolutions taken collectively by the nursing profession [11]. Chief amongst the resolutions was a need to develop a national policy intended to provide uniformity in the offering of nursing programmes. The specific focus areas included student recruitment, selection and admission; funding and support; clinical education and training of nurses; and non-alignment of nursing qualifications to the provisions of the NQF Act (Act 67 of 2008) [12].

The translation of the Nursing Compact into a National Policy for Nursing Education and Training became the logical point of departure. This process reiterated the diverse contextual realities of the nine South African provinces from a strengths-based perspective and the necessity of optimising resources for nursing education and training. An integrated approach to nursing education was proposed. A discussion of the cascade of processes followed to analyse the existing framework and propose elements for the policy follows.

## Research methodology

**Fig. 1:**
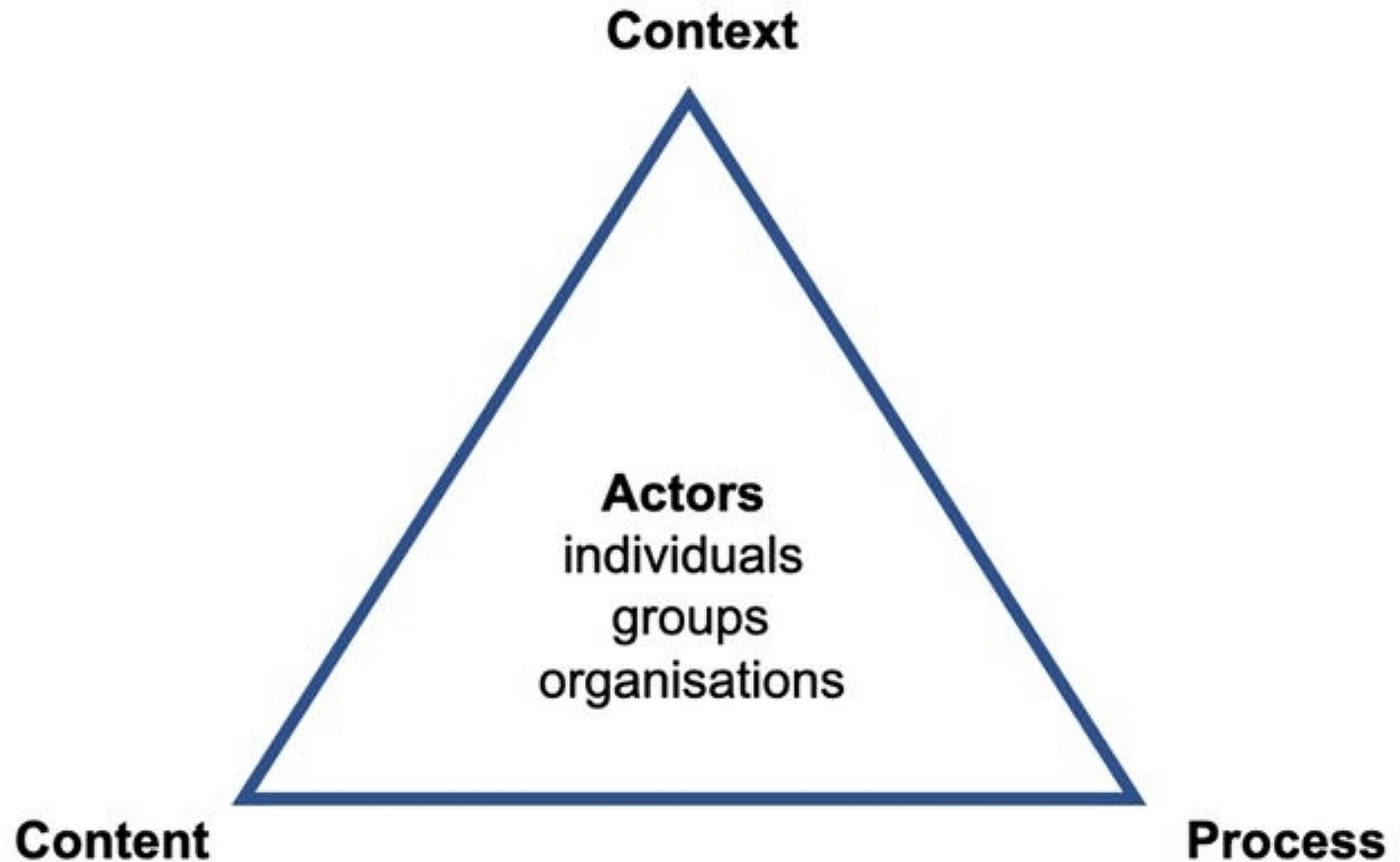
Walt and Gilson’s (1994) policy triangle analysis framework

The policy development process of Walt and Gilson’s Policy Triangle Analysis Framework [13], illustrated in Fig. 1 below, served as an analogy for analysing the framework to integrate nursing education into the higher education band. The Policy Triangle Analysis Framework considers the context, actor, content and process *components.* The context refers to the prevailing circumstances informing the policy; *actors* are key informants who were central in formulating and implementing the policy; *content* is about the policy elements; and *process* refers to how the policy is initiated, formulated, negotiated, communicated, implemented and evaluated [14]. This approach enabled a thorough examination of the various factors that could influence the policy development and implementation processes [15] and guided the authors toward more effective planning for successful execution.

### Context

Recent legal imperatives required alignment of all nursing education programmes to the higher education prescripts and health system demands. A national overarching framework became apparent; this framework was to ensure alignment of health service expectations and higher education prescripts. The context required developing a national policy to steer stakeholders through the transitional period, providing a directive for implementation. Thus, the policy would need to outline the basis for a uniform framework within which nursing education could be provided.

### Actors

Crucial to effective decision-making is understanding the characteristics of the diverse stakeholders (actors) involved in the policy development and implementation process. Stakeholders included those from the quadruple helix system (government, academia, industry and communities) and included government officials, nursing educators, healthcare professionals, students and other relevant parties. It was essential to gain a comprehensive understanding of their respective roles, interests and power dynamics to foster consensus and garner support for the proposed reforms.

**Fig. 2:**
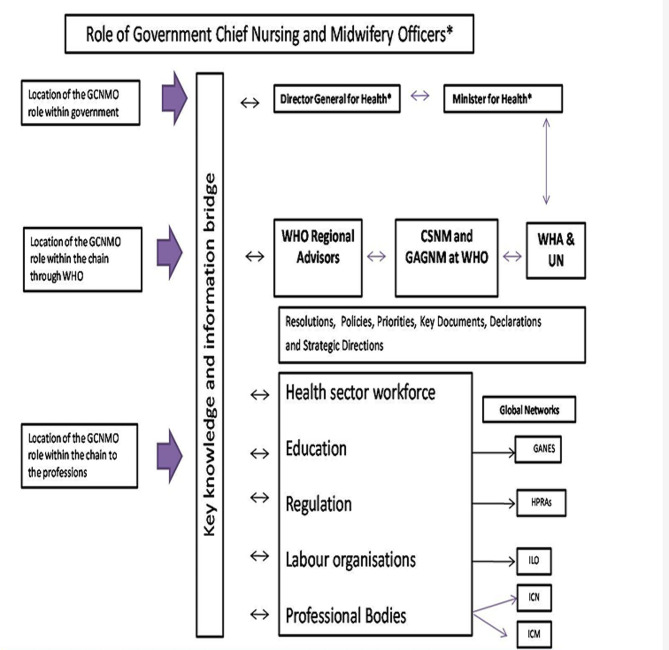
Role of government chief nursing and midwifery officers. (Source: WHO [5, 16])

Throughout the policy development journey, a reiterative and reciprocal stakeholder engagement process was conducted. Participants were actively engaged and were kept informed about the progress made at each stage. Engagement occurred through formal and informal consultations whereby input was received on policy proposals within specific thematic areas. Furthermore, these stakeholders critically reviewed and analysed the final draft policy for its fitness for purpose and adequacy of content. As the central figure for nursing in the country, the Chief Nursing Officer (CNO) led the process, solicited input and provided comprehensive oversight to ensure a well-informed policy. This was to be kept in line with the execution of the Government Chief Nursing and Midwifery Officers’ policy advice and responsibilities, as outlined by the World Health Organisation [5, 16]. See Fig. 2 below.

The following stakeholders played pivotal roles in the development of the nursing education and training policy:

- A Technical Working Group (TWG) was established that included experts in nursing education and practice to offer technical expertise and insights;
- The College Principals and Academic Staff Association (CPASSA) addressed disparities in nursing education and training;
- Provincial Directors for Nursing Practice contributed to the partnership between health establishments and nursing education institutions (NEIs) for clinical education;
- The National Department of Health’s (NDoH’s) Health Workforce Management and provincial counterparts in Human Resource Management and Organisational Development provided input on health workforce planning to align policy with national health strategies;
- The South African Nursing Council (SANC) guided the policy alignment with nursing competency frameworks and prepared new qualifications;
- The Forum for University Nursing Deans in South Africa (FUNDISA) shared experiences and insights about legacy nursing programmes in higher education;
- The private nursing sector ensured the inclusion of industry concerns within the policy framework;
- The Council on Higher Education (CHE) ensured policy alignment with qualification and programme requirements;
- The Department of Higher Education and Training (DHET) provided input on academic planning, student enrolment and monitoring, while the Joint Health Science Education Committee (JHSEC) coordinated and aligned health sciences education strategy and financing;
- The National Treasury advised on the financing model, which included a standardised bursary system and access to grants earmarked for health professions education;
- The Department of Planning, Monitoring and Evaluation in the office of the Presidency (DPME) assessed the policy’s alignment with the socioeconomic impact assessment and quality assurance frameworks.

Once the comprehensive consultation process was concluded, the policy was approved by the National Health Council (NHC), which is the highest decision-making structure for the health sector prescribed in the Health Act (Act 61 of 2003) [9].

### Process

**Fig. 3:**
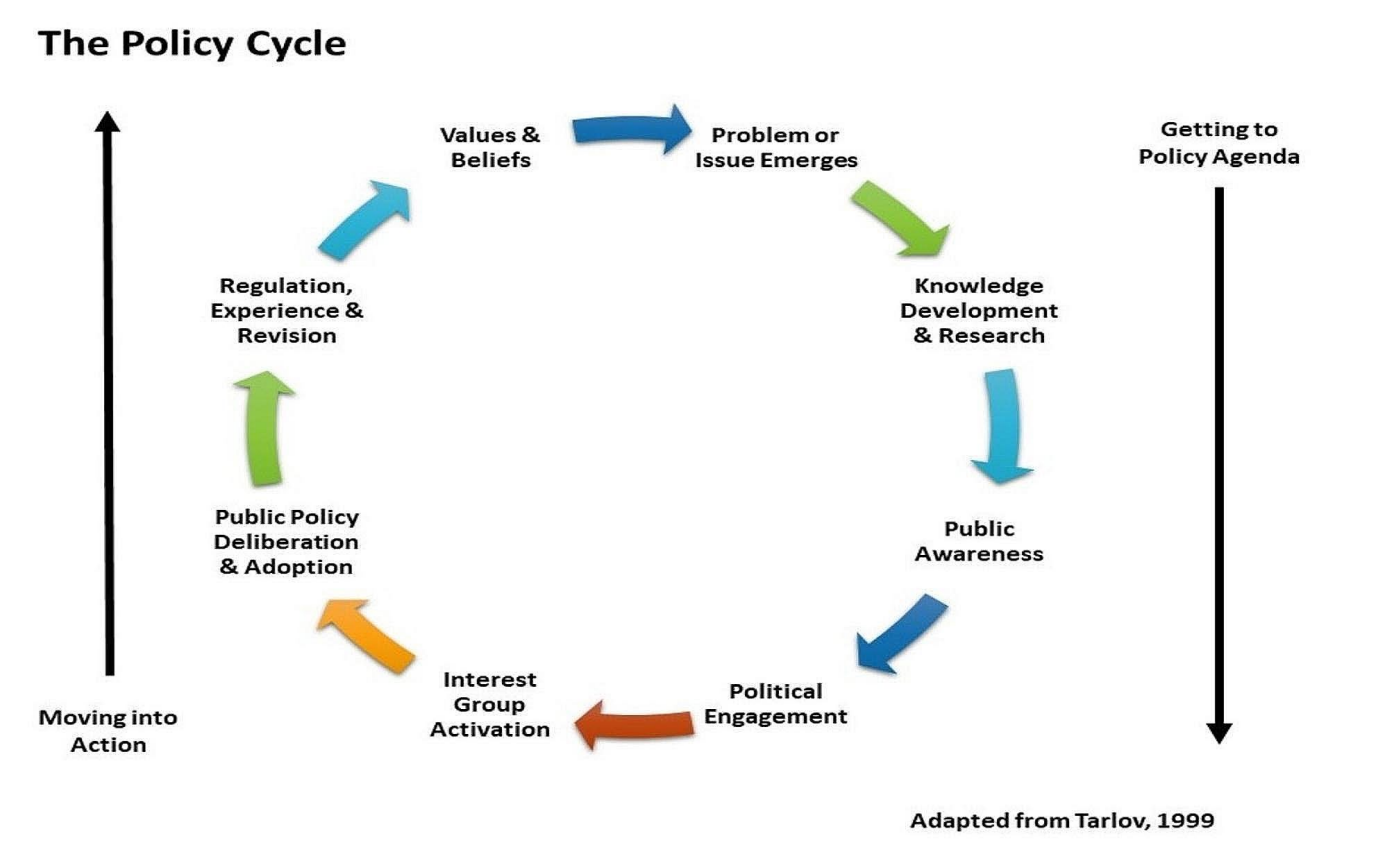
Adaptation of Tarlov’s public policy development process [20]

This stage entailed examining the policy formulation and implementation processes to help identify the stages, actors and steps involved in developing and enacting the policy [17]. The process phase of the policy framework analysis facilitated the identification of potential bottlenecks or areas that required improvement to ensure smooth implementation. Tarlov’s two-phase framework was employed [18] as depicted in Fig. 3. The framework is aligned with the government’s National Policy Development Framework [19], which facilitates the monitoring and evaluation of the policy.

#### Phase 1: Public consensus/national agenda building

##### Step 1: Values and beliefs

In this phase, values central to the provision of nursing education and training were adopted, including an education system informed by patient-centred care, safe nursing practice, evidence-based nursing practice, student-centred teaching and learning, professionalism, and service orientation and career focus. Whilst Tarlov’s model provides for values and beliefs as key tenants of the Public Consensus/National Agenda Building phase, this study specifically identified values as the only pertinent factor in this context.

##### Step 2: Problem or issue emerges

In this step, specific problems that needed to be addressed by the policy were identified. Firstly, prior to the advent of the new NQF, programmes leading to nursing qualifications were offered by diverse NEIs with varying settings, management and governance models. Inadvertently, NEIs’ different governance practices affected recruitment, selection, progression and articulation toward achieving full qualifications for prescribed nursing categories. Hence the need for a national policy to give effect to the provisions of the NQF within the context of nursing education.

Secondly, inadequate internal management systems led to overproduction of lower categories of nurses as elucidated by a survey conducted by the regulatory body [21]. This inadvertently perpetuated a mismatch between available nurses and service delivery demands. Furthermore, limited clinical training impeded the attainment of requisite competencies for professional registration [8, 22].

Thirdly, there was massification of production of nurses through two streams of one of the programmes. For instance, both colleges and universities produced registered nurses and midwives through separate degree and diploma programmes [23]. This resulted in service disharmony and hindered smooth articulation and transfer of credits between programmes. Key amongst these concerns was that midwifery training was accelerated [24, 25], which could potentially impact on student midwives’ competencies within their prescribed scope of practice.

The fourth problem related to the dual status of students in pre-registration nursing programmes. There was a lack of uniformity/standard position in the country concerning the status of the students in nursing and systems of managing students. For instance, college students were employees with full benefits, whilst university students had full student status. The net effect was misalignment between graduates’ skills and competencies and health service delivery requirements.

##### Step 3: Knowledge development and research

During this step relevant information related to the issues identified in Step 2 was gathered, including data, research findings, expert opinions, and any other insights that could contribute to a comprehensive understanding (knowledge) of the problems. A thorough analysis of available data and research findings was conducted to identify patterns, root causes, potential solutions, and the likely impact of different policy options. Simultaneously, several consultation workshops were held to engage key stakeholders. These consultation workshops covered activities such as consolidation, identification, review, collation, analysis, verification, brief development, and the establishment of a working group. Additionally, a composite thematic document was created and subjected to content validation through a think tank.

##### Step 4: Public awareness

Awareness campaigns were held with stakeholders central to policy implementation including academics, provincial heads of health, labour unions, regulatory bodies, higher education and senior management teams in provinces. Inputs from stakeholders were collated to produce the draft concept document. The background document was further improved based on input from the TWG. The National Stakeholder Consultative Meeting output was compiled into a composite document.

#### Phase 2: Political/public policy actions

##### Step 5: Political engagement

Cognisant of the implications of the policy to provincial structures responsible for quality, quantity, and relevance in nurse production, a focussed engagement was held with administrative Heads of Health (HoDs) in the nine provinces. During the workshops the policy was subjected to scrutiny by the HODs in preparation for brefing their Members of Executive Council (MECs). Specific focus was given to validation of policy for fitness for purpose, content accuracy and sufficiency. Given that MECs are political heads of provincial health departments and also members of the NHC - the highest decision-making body of the sector prescribed in the Act [9], a thorough briefing was essential to garner their support for policy approval by the NHC.

##### Step 6: Public policy deliberation and adoption

During this phase, the draft policy was subjected to NDoH statutory processes, including review by the National Department’s Legal Unit. The policy was declared to have met the socio-economic impact assessment (SEIAS) and quality assurance frameworks of the Department of Policy Monitoring and Evaluation (DPME). Conducting the SEIAS empowered policymakers and other decision makers to assess the potential effects of the proposed policy on diverse stakeholders and sectors to minimise any negative impacts. The policy was then finalised, and its approval was sought from the NHC.

##### Step 7: Interest group activation

The policy was presented to the NHC for adoption and subsequently submitted to the minister, who signed the policy for final approval on 31 January 2019. The policy was gazetted on 05 April 2019. The Minister conducted a policy launch event to introduce the policy to the relevant stakeholders.

##### Step 8: Regulation, experience and revision

**Fig. 4:**
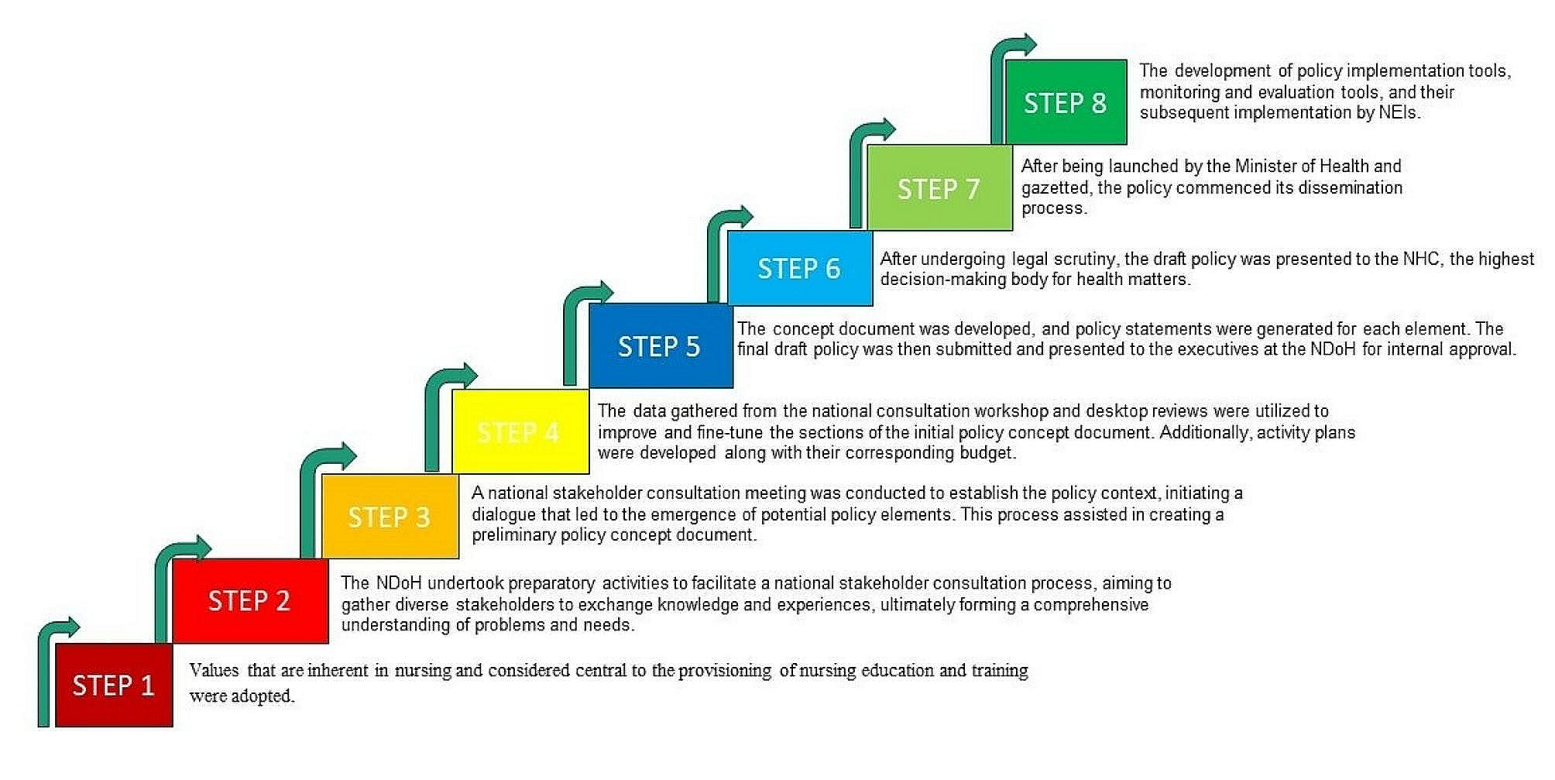
Nursing education policy framework development process

NEIs began implementing the approved policy. Policy implementation tools (guidelines) were developed. The Office of the CNO began conducting supportive visits to evaluate the implementation of the policy and assess its effectiveness. The steps followed in developing the policy are summarised in Fig. 4.

### Content

The rationale for developing the policy was to ensure a unified system of nursing education and training:

To address the evolving healthcare landscape, there is a need for a national framework that is aligned with health service demands and sound education principles. This framework can guide NEIs to prepare to offer higher education qualification subframework (HEQSF)– aligned nursing programmes.

The policy has broad elements that are designed around challenges in the provisioning of of nursing education programmes. Its primary objective is to establish a consistent framework for nursing education that enables smooth transitions between different nursing programmes. Furthermore, the policy aims to enhance the quality and alignment of nursing education with professional registration requirements. It serves as a national guideline for locating nursing educationwithin a post-school education system. The key elements include the following:

- Implementing a standardised system to attract a diverse range of students to nursing programmes;
- Ensuring the competency and capability of the nursing and midwifery workforce; and
- Enhancing the synchronisation and standardisation of nursing education and training through strengthened clinical training platforms.

## Discussion

The policy ensures alignment of higher education prescripts to health service demands by providing a framework that can guide NEIs to prepare to offer higher education qualification subframework (HEQSF)-aligned nursing programmes. Thus, expanding access, improving quality and diversifying the provision of nursing education and training through policy imperatives discussed below.

### Uniformly promote compliance with all legislative requirements for provisioning of nursing programmes

#### Accreditation and registration

All NEIs, both public and private, along with their programmes, must be accredited by the SANC and CHE and registered with the SAQA. Accreditation and registration need to be aligned with applicable legislation as prerequisites for offering programmes in the HEQSF so that nursing qualifications can be recognised and registered by the SANC.

#### Optimizing students’ fitness for practice

Full student status must be retained for the entire study period. Registration with the SANC should occur within a specified timeframe after admission to an NEI. Evolving educational landscapes should take into consideration the characteristics and needs of the student population.

### A standardised system to attract and recruit diverse students into nursing programmes, with a uniform recruitment and selection framework and progression across nursing programmes

#### Entry, admission and selection requirements

Three entry-level programmes designed as standalone qualifications determine access to nursing programmes. These entry levels can either be at Auxiliary, Diploma or Bachelor programmes.

The NDoH developed an implementation guideline for standardised recruitment, selection and admission of students to new nursing programmes. This ensures standardised recruitment, selection and admission procedures for individuals with NEIs. These guidelines aim to guarantee that the actions and decisions of NEIs, which affect the quality, quantity and relevance of the future nursing workforce, are equity-minded and socially accountable to priority health continuum problems and service needs of the communities and regions served by education institutions.

#### Provision for experiential knowledge

Students with prior experience in the nursing/midwifery context and who acquire knowledge through the workplace and other learning settings can have their prior learning recognised. National policies that underpin experiential based kniowledge are implemented accordingly.

#### Career pathways

**Fig. 5:**
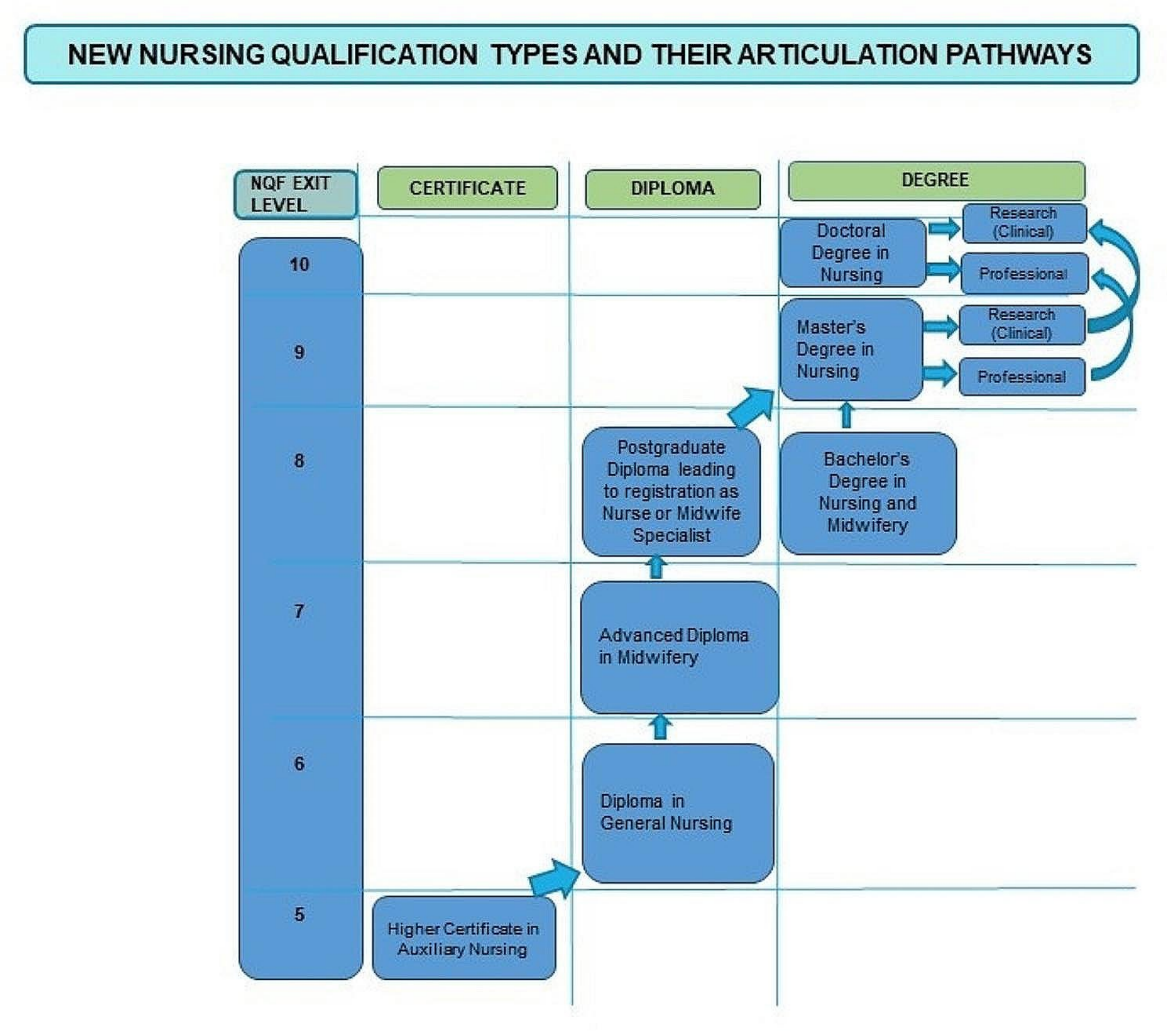
Career pathways for nursing (adopted from Bezuidenhout et al. [27]) and SANC, 2016 [28]

In line with the NQF, the policy provides for seamless, predictable and coherent career pathways within the nursing qualifications. After a lengthy and protracted processes over a period of several years, involving collaboration between the SANC and critical partners [26, 27], nursing qualifications were eventually aligned to the NQF as depicted in Fig. 5.

Although the policy provides provisions for the introduction of new clinical programmes at the NQF Level 7 in a phased-in approach, there have not been any additional clinical programmes at this level. In addition to the current midwifery programme, the need for other advanced diplomas should be explored, and additional clinical programmes **should** align with health service needs and disease burdens.

### A synchronised and standardised system for clinical training through the strengthening of clinical platforms

#### Clinical teaching and learning

The health authority retains overall coordination of clinical teaching and learning to optimize standardisation. Accordingly, a framework for collaborative action in the form of a memorandum of understanding (MOU) between the health authority and NEIs has been developed. The MOU stipulates all institutional arrangements and resources requisite to optimize clinical learning experience of students.

### Synchronised and collaborative partnerships between the two departments (NDoH and DHET) and their respective councils (SANC and CHE)

The NDoH and DHET collaborated to define a legal framework to guide transitional arrangements that have been put in place to ensure that nursing colleges can continue to operate until they are declared as higher education colleges. This also involves creating an enabling environment for the accreditation of new qualifications on the HEQSF, registration of new qualifications on the NQF, recognition of new programmes by the SANC and enabling the public nursing colleges to market and enrol students on the new qualifications.

### A uniform and standardised financing mechanism for nursing education and training

A costing exercise was conducted to determine the uniformity of the provisioning and management of nursing education and training and to operationalise the policy within the requirements of higher education and the regulatory framework. Ultimately, it was concluded that there was a need to leverage available funding streams (intersectoral, interprofessional and interprovincial) to promote access to and optimise nursing education resources [8]. Although nursing colleges have been re-established as providers of post-school education programmes, they remain funded by the provincial fiscus. Invariably, this institutional type cannot tap into established funding earmarked for post-school education programmes. Thus, there is a need to align funding for programmes offered by colleges with a funding framework for the post-school education funding model.

## Conclusion

The policy served as a key impetus for accelerating the process of repositioning NEIs within the post-school education system. It serves as an instrument for nursing education and training to function within both healthcare and higher education policy frameworks.

The inclusion and active involvement of clinical nurse leaders in the policy development process, particularly as members of the TWG, has enhanced the quality and applicability of teaching and learning, both in classroom and clinical settings. This collaboration between nursing education and clinical services continued through the development of policy implementation tools and resulted in enhanced acceptance and adherence to the tools and promotion of a seamless transition for nursing students from education to practice/service. Alignment of the policy with the government’s National Policy Development Framework enhances monitoring and evaluation efforts. Insights and lessons gained from the policy development process and stakeholder engagement, along with the resulting policy imperatives, can be leveraged to expedite the integration of other related health sciences professions into higher education. This approach will ensure a continuous supply of professionals with the necessary skill mix to support a responsive healthcare system.

## Data Availability

All data generated or analysed during this study are included in this published article.

## Abbreviations

CNO: Chief Nursing Officer
CPASSA: College Principals and Academic Staff Association
CHE: Council on Higher Education
DHET: Department of Higher Education and Training
DPME: Department of Planning, Monitoring and Evaluation in the Presidency
FUNDISA: Forum for University Nursing Deans in South Africa
HEQSF: Higher Education Qualification Subframework
JHSEC: Joint Health Science Education Committee
MEC: Member of Executive Committee
MOU: Memorandum of Understanding
NDoH: National Department of Health
NHC: National Health Council
NQF: National Qualifications Framework
NEI: Nursing Education Institution
SANC: South African Nursing Council
SAQA: South African Qualifications Authority
SEIAS: Socioeconomic Impact Assessment
TWG: Technical Working Group
WHO: World Health Organisation
